# Emotional distress in the early stages of the COVID-19 related lockdowns depending on the severity of the pandemic and emergency measures: a comparative online-survey in Germany, Austria and Italy

**DOI:** 10.1186/s12888-021-03505-7

**Published:** 2021-10-15

**Authors:** Christiane Eichenberg, Martin Grossfurthner, Sibylle Kietaibl, Greta Riboli, Rosita Borlimi, Stefana Holocher-Benetka

**Affiliations:** 1 Institute of Psychosomatics, Faculty of Medicine, Sigmund Freud University, Vienna, Austria; 2 Faculty of Psychology, Sigmund Freud University, Vienna, Austria; 3 Faculty of Medicine, Sigmund Freud University, Vienna, Austria: Evangelical Hospital Vienna, Vienna, Austria; 4 Faculty of Psychology, Sigmund Freud University, Milan, Italy

**Keywords:** COVID-19, Lockdown, Emergency measures, Online survey

## Abstract

**Background:**

The first wave of the COVID-19-pandemic hit different countries with varying degrees of severity, so that differences in the type and level of emergency measures were also necessary. It can be assumed that the psychological burden was higher in countries subjected to a more severe course of the pandemic (Italy) than in countries subjected to a less severe one (Germany, Austria).

**Objective:**

To investigate and contrast the wellbeing of the population in Italy, Austria, and Germany in the early phase of the first lockdown.

**Method:**

Online survey on *N* = 4289 individuals. The questionnaire comprised a self-administered section, exploring the dimensions: perceived severity of COVID-19, perceived risk of disease, concerns related to COVID-19, emergency measure acceptance and emotional distress due to emergency measures; and standardized scales to record emotional state and coping: Stress-Coping-Style Questionnaire, Positive and Negative Affect Schedule, State-Trait-Anxiety-Inventory.

**Results:**

The three countries displayed significant differences in all investigated dimensions (*p* < .001). Italian participants assessed the COVID-19 virus as much more dangerous (*p* < .001), but despite the prevalence of the virus, the subjective risk of disease was perceived to be lower in Italy (*p* < .001). This could be a positive effect of the restrictive curfews set by the government in Italy. The emergency measures were generally perceived to be very effective in all three countries, but due to the duration and the severity of the measures, the fear and stress-reaction were the strongest among Italian participants (*p* < .001).

**Conclusion:**

The stricter measures in Italy prevented an application of many positive stress processing strategies, which, in turn, fostered the perpetuation of stresses and fear.

**Supplementary Information:**

The online version contains supplementary material available at 10.1186/s12888-021-03505-7.

## Introduction

Since the outbreak of the COVID-19 crisis, severe medical challenges, as well as their mental and social consequences, determine public life. The world is confronted with a pandemic of global scope. Worries and fears during this crisis are shaped individually and concern various aspects of life, including health, employment, and social relations [1]. The first lockdown – which started on 08.03.2020 in Italy, on 16.03.2020 in Austria, and on 23.03.2020 in Germany, and lasted 7 weeks in Austria and Germany and 10 weeks in Italy – constituted a unique situation. It was followed by a phase of an incremental loosening of the emergency measures. However, “normality” cannot be expected in the near future. The infection rates have been rising again in many countries, including Italy, Austria, and Germany, since August 2020 leading to a new lockdown in these countries from the beginning of November 2020 onwards. So far, the course of the COVID-19 pandemic has been cyclical, characterized through highly acute phases and phases of moderate infection rates. Each phase was accompanied by respective statutory emergency measures. In order to assess the psychosocial impacts of the pandemic on the overall population in general as well as on vulnerable groups in particular, it is necessary to conduct studies that capture these stresses in different phases of the pandemic. The lockdown thereby constitutes a particular phase with the most severe social restrictions.

### Current state of research on the psychological impacts of the lockdown

The research activity on COVID-19 related questions was enormous within all disciplines able to contribute. This is illustrated by a data inquiry of “PubMed” for the medical field. In June 2021, the search term “COVID” lead to more than 138.000 hits. If narrowed down to “COVID & lockdown”, more than 6.500 hits were attained at the same point in time. The very specific search for “COVID & lockdown & psychological impact” still lead to 600 results. It is thus no surprise that there already exist meta-analyses summarizing the results of studies on the psychological stresses within the respective countries e.g. for China see [2]. These 600 publications do not only comprise country-specific studies, on countries like Italy [3–5], Spain [6], Nepal [7], China [8], Africa [9], and India [10], but also cross-sectional studies accounting for mental impacts on the overall population e.g. [11, 12] as well as on specific socio-demographic groups such as students [13], vulnerable groups like the elderly [14] and doctors (e.g. [15] for Italy), or clinical groups, like psychiatric patients e.g. for Italy [16] or children and adolescents with ADHD [17]. Longitudinal studies looked at the prevalence of mental disorders before and during the lockdown (see 11 for the United Kingdom), while retrospective studies analyzed in how far and what kinds of suicides that took place during the lockdown, can be traced back to this peculiar situation [18].

The studies can be further differentiated with regards to their outcome measures and the moderating variables included. They measure, for example, clinical stresses such as fear, depression, or psycho-traumatic symptoms as well as health-psychological variables like the impacts on sexual activity [19] or online gambling behavior as a coping mechanism [20].

Moderating variables were included to identify certain risks and protective factors, operationalized, e.g., through attachment style and affective temperament [4]. Genetic influencing factors were compared to environmental influences using twin studies [21]. Besides questionnaires, psycholinguistic methods were used, like in the study by Su et al. [22], which analyzed the changes in psycholinguistic trades of social media posts before and after the lockdown in Wuhan (analyses of posts on Weibo) and Lombardy (analysis of posts on Twitter). Thus, cross-country comparisons were conducted to detect potential culturally specific ways of dealing with a lockdown. However, according to our knowledge, there exists no study assessing the emotional wellbeing in as well as the resilience to the pandemic crisis within the population of different countries experiencing the pandemic to varying degrees of severity and consequently facing different lockdown phases. In a European comparison, Italy was hit the hardest in the first phase of the pandemic, counting 3405 COVID-19 related deaths on 19.03.2020 (https://opendathunadpc.maps.arcgis.com/apps/opsdashboard/index.html#/b0c68bce2cce478e), and thus for the first time more victims than China. At the same point in time, there were only six deaths in Austria (Official figures from the Federal Ministry of Social Affairs, Health, Care and Consumer Protection of Austria: https://coronatracker.at), and 20 in Germany (Official figures from the Robert Koch Institute: https://www.rki.de/DE/Content/InfAZ/N/Neuartiges_Coronavirus/Situationsberichte/2020-03-19-de.pdf?__blob=publicationFile).

Due to the degree of severity with which Italy was affected, the lockdown was not only in plaece for longer, its measures were also more restrictive than the ones implemented in Germany and Austria. The people in these countries not only continued to be allowed to leave their apartments for work-related reasons, people in Germany could, for example, also do sports with another member of the same household (see the German SARS-CoV-2 regulation on containment measures from March 17^th^, 2020 of the federal state of Berlin: https://www.berlin.de/sen/justiz/service/gesetze-und-verordnungen/2020/ausgabe-nr-12-vom). Likewise, the house could be left to cover basic needs, including mental and physical recreation in Austria (https://www.lbg.at/servicecenter/lbg_steuertipps_praxis/corona_virus_bundesweit_einheitliche_verkehrsbeschränkungen_ab_16_märz_bis_22_märz_2020/index_ger.html). In contrast, the measures set in Italy were much more far-reaching. At the time of our study, the population was only permitted to leave the house for the following reasons: work-related reasons if working within a system-relevant job, individual walks no further than 200 meters away from the place of residency, going to the hospital or visiting a doctor in cases of emergency, shopping in open supermarkets and essential shops (e.g., pharmacies, tobacco shops) (http://www.governo.it/it/articolo/coronavirus-firmato-il-dpcm-22-marzo-2020/14363).

We conducted an online survey in the early phase of the first lockdown in Italy, Germany, and Austria in order to contrast the emotional wellbeing of the population of these countries. We aimed at capturing the perceived danger of the virus, the specific concerns and stresses as well as the resilience of the populations in the respective country. Likewise, the usage of apps for compensatory stress management, as well as a potential change in attitudes toward social media as a means of communication with family and friends, were surveyed.

We hypothesize that within the Italian population – the population of a country, that experienced the pandemic very severely and consequently implemented very restrictive emergency measures – the virus is not only assessed to be more dangerous but also the stresses in terms of worries, fear, and negative emotions are higher than among the Austrian and German population. Due to the considerably more restrictive measures, we additionally assume, that in Italy digital media is used to a larger extent for compensatory stress management (e.g., mental health apps) and that – as in this country no social-physical contacts were possible – social media was used as a social compensation. This could cause a change of attitudes towards these media.

We thus contrasted the Italian with the German and Austrian population in respect to the following questions:

- How dangerous is the virus perceived to be?

- How high is the subjective risk of disease perceived in dependency on the emergency measures?

- How does the acceptance of the statutory emergency measures develop and which emotions accompany the respective measures?

- Which effects do the statutory measures have on fear and emotional wellbeing of the population?

- Which coping strategies can become effective under the respective measures?

## Materials and method

### Study design

The Austrian, German, and Italian populations were invited to participate in an online survey via social media and newspapers. We used the online survey SoSci (https://www.soscisurvey.de) for data collection. A pre-test with ten participants allowed us to interpret the results in order to improve feasibility, intelligibility, and comprehensiveness. The survey was available online from March 22 to 29 (beginning of the lockdown in Italy: March 8^th^, 2020; beginning of the lockdown in Austria: March 16^th^, 2020; beginning of the lockdown in Germany: March 23^rd^, 2020). Participants received information about study design and data protection before filling in the questionnaire. The duration of the questionnaire was about 25 minutes. The Ethics Commission at Sigmund Freud University Vienna approved this study (date of approval: March 18^th^, 2020).

### Instruments

Participants filled in a self-administered questionnaire, which included socio-demographic data and various sets of questions concerning the COVID-19-pandemic (see Appendix)

### Socio-demographic data

Besides information on gender (male/female/diverse), age (in years; retrospectively subsumed in 6 groups: 18-29/30-39/40-49/50-59/60-69/ 70 + year-old), and highest educational level (7 categories from “no graduation” to “university degree”), we asked for annual income (up to 25.000 €/25.000-40.000 €/40.000-70.000 €/70.000 -100.000 €/ > 100.000 €) and the number of people per household.

### Covid-19-pandemic questionnaire

*Perceived severity of COVID-19:* 2 items (5-point Likert scale). The internal consistency (Cronbach's alpha) in our sample is α = .65.

*Perceived risk of disease:* 3 items on the danger of the virus itself, the risk of becoming ill, and the risk of transmitting the infection COVID-19 (5-point Likert scale). The internal consistency (Cronbach's alpha) in our sample is α = .60.

*Emotional distress due to emergency measures:* 10 items on negative feelings relateed to the behavioral measures (5-point Likert scale). The internal consistency (Cronbach's alpha) in our sample is α = .87.

*Emergency measure acceptance:*15 items on the assessment of the value and efficacy of behavioral measures: self-isolation, quarantine, traveling restrictions, smart working, cancellation of events (5-level Likert scale). The internal consistency (Cronbach's alpha) in our sample is α = .90.

*Concerns related to the COVID-19-pandemic:* 8 items on concerns about health, society, and economy (5-point Likert scale). The internal consistency (Cronbach's alpha) in our sample is α = .64.

*E-mental health usage:* 4 items on app-usage for stress management, overcoming fears, socializing, and other (dichotomy); one question on the change of attitudes towards social media. The internal consistency (Cronbach's alpha) in our sample is α = .53.

We used standardized scales in the following order to capture current emotional wellbeing (affects, fear) and possibilities of stress management (coping styles):

Assessment of current emotional wellbeing: We adapted the German version of the *Positive and Negative Affect Schedule* - *PANAS* from the one developed in 1988 by Watson, Clark, and Tellegen [23] to measure emotional states. It consists of twenty adjectives that describe different emotions and feelings. The two groups of ten terms are accurate markers of either positive or negative affect, and subjects assess their intensity on a five-point scale from "not at all" to "extremely". The internal consistencies (Cronbach's alpha) for both subscales are α > .84. To design a questionnaire of reasonable length, we only included the items on negative affect.

Assessment of stress management strategies: *Stress Coping Style Questionnaire - SVF 78* [24]: This questionnaire evaluates coping styles and processing patterns in stressful situations. It is composed of 13 subscales, each describing reactions to stress in terms of time and situation-stable (stressor) personal characteristics. The internal consistencies (Cronbach's alpha) of the SVF subtests result are between α = .77 and α = .94.

Assessment of current anxiety: *State-Trait Anxiety Inventory - STAI* [25]: This is a standard tool in anxiety and stress research consisting of two subscales incorporating 20 items (4- points Likert scale) to differentiate anxiety as a state and as a trait. The internal consistency for both subscales is α = .90. Since we were only interested in the current condition, we only used the state-scale.

### Participants

6334 Austrians, Germans, and Italians registered via the dedicated link during the survey validity period. 12.55% of the participants (*N* = 795) did not complete the first page, on which participants were informed on the content of the study as well as on data processing and asked to give written consent to the participation in the study. Only about 5.89% (*N* = 294) of Austrians and Germans and 4.67% (*N* = 62) of Italians abandoned the questionnaire after the second page, on which socio-demographic data was collected. The overall dropout rate of 32.29% (*N* = 2045) is acceptable [26]. The actual sample size, after excluding all invalid cases or dropouts, consisted of *N*= 4289 data sets (Germany: n = 704, Austria: n = 2359, Italy: n = 1226) and was therefore included in the evaluation.

### Statistical analysis

We used the Statistical Package for the Social Sciences Program (SPSS Version 24) for data input, processing, and statistical analyses.

First, we performed goodness-of-fit tests to get an overview of the data. The distribution of all questionnaire-scores deviated in at least one of each group significantly from normality (Kolmogorov-Smirnov and Shapiro-Wilk < .05). As a consequence, we performed Kruskal Wallis Tests, followed by pairwise Post-hoc-Tests to compare the COVID-19 scales *Perceived risk of disease*, *Perceived severity of COVID-19*, *Emergency measure acceptance,* and *Emotional distress due to emergency measures* between age and income groups. It was of interest whether sociodemographic variables have an influence on the assessed questionnaire scales independently of the country affiliation and might thus influence the results of the cross-country comparison.

Finally, Kruskal-Wallis Tests, followed by post-hoc-tests, were performed to compare scores of the COVDI-19-scales and scores of the *PANAS-, STAI-* and *SVF78-*scales between the three countries.

Over a short period of time, we generated a much larger sample than would have been necessary according to the sample size calculation. With three groups to be tested for differences with respect to an interval-scaled variable (Anova recurrent), the calculation with G*Power (Heinrich-Heine-University Düsseldorf; https://www.psychologie.hhu.de/arbeitsgruppen/allgemeine-psychologie-und-arbeitspsychologie/gpower) yielded a sample size of approximately 260 subjects. Larger sample sizes produce more reliable results with greater precision and explanatory power through avoiding a Type I error [27]. Particularly with regards to the novelty of the study in connection with a topic that has not yet been researched, such a large sample also has the advantage of being able to detect even the smallest differences and effects. The disadvantage, however, is that even unimportant, minimal differences may be interpreted as significant. Therefore, in order to test the reliability of our results, we drew a random theoretic sample consisting of only 262 subjects from the existing population. We took care to ensure that the socio-demographic variables of age, gender, and education were distributed as evenly as possible. On this random theoretical sample, we then again performed goodness-of-fit tests to get an overview of the data. As a result, the distribution of all questionnaire-scores deviated in at least one of each group significantly from normality (Kolmogorov-Smirnov and Shapiro-Wilk < .05), except the SVF78-scales and the scale Concerns related to COVID 19. We then performed Kruskal Wallis Tests, followed by pairwise Post-hoc-Tests, and Browne-Forsythe Tests, followed by Bonferroni Post-hoc-Tests, to compare the scores of the PANAS-, STAI- and SVF78- as well as the COVID-19 scales between the three countries.

## Results

### Demographics

Within the total sample of *N* = 4289 subjects, the gender distribution shows a higher proportion of women with 2911 female respondents (68%) versus 1332 male participants (31%) and 46 subjects (1%) who did not specify their gender. The average age was 35.2 years (*SD* = 12.04). The distribution of the socio-demographic variable “highest educational level” also shows that the sample at hand has an unusually high level of education: 35.8% had a general qualification for university entrance, 60.2% a university degree. A total of 783 respondents declared living alone (18.3%), while the others were cohabiting, for the most part in a two-person household (2345 people or 54.7%). Approximately a quarter of the participants live with one or more children.

The three countries (Italy, Austria, Germany) exhibit an even distribution with regards to educational level (χ^2^ (2) = 3.06, p = .217) and gender (χ^2^ (2) = 1.06, p = .209). There is a significant difference regarding age (χ^2^ (2) = 20.87, p < .001). The Austrian sample contains significantly younger participants than the Italian sample. With regards to income, all three countries differ significantly (χ^2^ (4) = 168.21, p < .001), the annual income being the highest in Germany and the lowest in Italy. This is in accordance with statistics on the average gross monthly earnings, which are significantly lower in Italy than in the other two countries (e.g., https://de.statista.com/statistik/daten/studie/183571/umfrage/bruttomonatsverdienst-in-der.eu/).

The random theoretical sample consisted of 111 female (44%) and 141 male (56%) participants of which 21% were 18–29, 26.6% 30–39, 21.4% 40–49, and 7.5% 50–59 years old, while 13.5% were older than 60. The average age was 41.9 (*SD* = 14.33; *Md* = 40). Within the sample, 38.9% had a general qualification for university entrance, 38.9% a university degree, and 22.2% had neither the qualification for university entrance nor a university degree.

### COVID-19 questionnaire scores in socio-demographic comparison

The virus is perceived as dangerous in the whole sample (*N* = 4289). The *Perceived severity of COVID-19* median value is 8 out of 10 (*M* = 8.22; *SD* = 1.47). Kruskal-Wallis tests results show significant differences between the different age groups (χ^2^ (4) = 151.47, p < .001), Mann-Whitney tests prove differences between the age-groups of 50+ year-olds and all younger groups (18-49 years). Older people perceive the virus as significantly more dangerous. The situation is different in the assessment of the *Perceived risk of disease* (*M* = 8.58; *SD* = 2.34*)*.

Here too, significant differences between age groups appear (χ^2^ (4) = 36.97, p < .001). However, thee post-hoc-tests show that the two youngest groups (18-29 years and 30-39 years) report a significantly higher perceived risk of disease than all others. Within the different income groups and between genders no significant differences regarding the perceived danger of COVID-19 and the subjective risk of disease can be detected (*Danger of Covid-19*: income (χ^2^ (4) = 8.95, p = .062), gender: (χ^2^ (2) = 1.54, p = .462); *Subjective risk of disease*: income: χ^2^ (4) = 5.57, p = .234; gender: χ^2^ (2) = 3.91, p = .142).

Emergency measures implemented by the Austrian, German and Italian governments are considered to be highly effective. The *Acceptance* median value is 72 out of 75 (*M* = 69.76; *SD* = 6.42), revealing a high level of endorsement. Kruskal-Wallis tests results show no significant differences between the different age groups (χ^2^ (4) = 2.77, p = .596). Regarding the income groups, differences can be found (χ^2^ (4) = 14.93, p = .005). The Mann-

Whitney tests prove differences between low- and high-income groups, the former showing a lower level of acceptance. Furthermore, gender differences can be found (χ^2^ (2) = 36.47, p < .001). Post-hoc Mann-Whitney tests show that women ascribe more acceptance to the measures than men (z = - 5.70, p < .001). Besides the broad approval of the governments' action, the level of discomfort stemming from the restrictive measures (*Emotional distress due to emergency measures*) remains relatively low. A skewed distribution is observed, with a median of 14 out of 50 (*M* = 16.34; *SD* = 6.68). Nevertheless, this essentially positive picture does not apply to all groups to the same extent. The Kruskal-Wallis test shows different levels of emotional distress between different age (χ^2^ (4) = 31.97, p < .001), income (χ^2^ (4) = 31.97, p < .001) and gender groups (χ^2^ (2) = 32.28, p < .001). As post-hoc tests illustrate, the group of 50+ year-old, men and high-income groups show less discomfort than all other groups.

### Cross-country comparison

The three countries displayed significant differences (p < .001) in all dimensions derived from the STAI, SVF78, PANAS, and COVID-19 questionnaires (see Table 1) in the total sample (*N* = 4289).

**Table 1:** Descriptive Statistics and Kruskal-Wallis Tests results for emotional state, coping and COVID-19-scales, *N* = 4289

Scales		Italy (*n* = 1226)	Austria (*n* = 2359)	Germany (*n* = 704)	*p*	*χ*^*2*^
*STAI State Anxiety*	*M*	49.54	43.45	44.50	< .001	407.56
	*MD*	50	42	43		
	*SD*	3.91	11.90	12.15		
*PANAS Negative Affect*	*M*	26.72	19.88	21.35	< .001	883.94
	*MD*	27	19	20.5		
	*SD*	5.28	6.25	6.62		
*SVF78 Positive Stress Behavior*	*M*	12.47	18.91	18.95	< .001	2367.44
	*MD*	12.57	19	19.07		
	*SD*	1.21	2.87	2.82		
*SVF78 Negative Stress Behavior*	*M*	12.93	16.37	16.32	< .001	643.24
	*MD*	13	16.25	16		
	*SD*	1.80	4.57	4.70		
*Concerns related to COVID-19*	*M*	27.61	25.22	26.15	< .001	175.28
	*MD*	27	25	26		
	*SD*	3.39	5.67	5.79		
*Subsale: Concerns about health*	*M*	3.05	2.04	2.17	< .001	445.58
	*MD*	3	2	2		
	*SD*	1.43	.91	3.05		
*Subscale: Concerns about financial problems*	*M*	3.91	2.12	2.17	< .001	1346.57
	*MD*	4	2	2		
	*SD*	1.06	1.14	1.19		
*Subscale: Concerns about the economic impact*	*M*	3.69	3.22	3.36	< .001	151.34
	*MD*	4	3	3		
	*SD*	1.23	1.16	1.16		
*Perceived Severity of COVID-19*	*M*	8.65	8.06	8.00	< .001	151.95
	*MD*	9	8	9		
	*SD*	1.31	1.47	1.57		
*Perceived Risk of Disease*	*M*	8.22	8.59	9.18	< .001	72.28
	*MD*	8	8	9		
	*SD*	2.24	2.36	2.33		
*Emergency measure acceptance*	*M*	69.50	70.43	67.99	< .001	205.17
	*MD*	71	73	71		
	*SD*	4.72	6.43	8.29		
*Emotional distress due to emergency measures*	*M*	21.63	13.86	15.46	< .001	1352.07
	*MD*	20	12	13		
	*SD*	6.60	4.94	6.68		
*App-Usage*	*M*	1.06	.34	.27	< .001	1324.43
	*MD*	1	0	0		
	*SD*	.65	.64	.62		

The results of the pairwise post-hoc tests are shown in Table 2. On the anxiety scale, Italian participants portray significantly higher values than Austrian and German participants. Besides higher anxiety levels, the Italian sample shows higher negative affects. The Austrian and German samples also differ significantly in this regard, with Austrian participants showing the least negative affect. With regards to stress management strategies, the Italian participants show significantly lower positive but also significantly lower negative stress management strategies compared to the other two countries. The Austrian and the German samples do not differ in this regard.

**Table 2:** Mann-Whitney-U-Test post hoc tests, *N* = 4289

Measures	group	group	*p*	*r*
*STAI State Anxiety*	Italy	Austria	< .001	.33
	Italy	Germany	< .001	.29
	Austria	Germany	.049	
*PANAS Negative Affect*	Italy	Austria	< .001	.49
	Italy	Germany	< .001	.40
	Austria	Germany	< .001	.10
*SVF78 Positive Stress Behavior*	Italy	Austria	< .001	.78
	Italy	Germany	< .001	.80
	Austria	Germany	.761	
*SVF78 Negative Stress Behavior*	Italy	Austria	< .001	.41
	Italy	Germany	<.001	.80
	Austria	Germany	.587	
*Concerns related to COVID-19*	Italy	Austria	< .001	.22
	Italy	Germany	< .001	.14
	Austria	Germany	< .001	.07
*Perceived Severity of COVID-19*	Italy	Austria	< .001	.20
	Italy	Germany	< .001	.21
	Austria	Germany	.631	
*Perceived Risk of Disease*	Italy	Austria	< .001	.07
	Italy	Germany	< .001	.20
	Austria	Germany	< .001	.11
*Emergency measure acceptance*	Italy	Austria	< .001	.23
	Italy	Germany	.434	
	Austria	Germany	< .001	.15
*Emotional distress due to emergency measures*	Italy	Austria	< .001	.61
	Italy	Germany	< .001	.50
	Austria	Germany	< .001	.10

Effect sizes: r = .10 small, r = .30 medium, r = .50 large (Cohen, 1988)

Italian respondents have significantly higher values on the COVID-19 scale *Perceived severity* than Austrian and German respondents. They thus perceive the virus as more dangerous. All three samples differ significantly from each other regarding the *Perceived risk of disease*. Accordingly, Germans report the highest, Austrians the second-highest, and Italians the lowest subjective risk of disease. The three populations also differ significantly with regards to the *Emergency measure acceptance* scale. The measures are most accepted by Austrians, followed by Italians and lastly Germans. For *Emotional distress due to Emergency measures,* Italians report the highest and Austrians the lowest values. The three countries also differ significantly regarding *Concerns related to the COVID-19-pandemic.* Again, Italians show the highest and Austrians the lowest values. Item analysis shows, compared to the Austrian and German sample, Italian participants report a higher concern about their health, and higher concern about financial problems, and the economic impact of the pandemic.

Regarding the use of mental health apps, Italian participants report a significantly higher usage than participants in the other two countries. Further, attitudes towards social media have considerably changed in Italy since the start of the pandemic. 70% of the Italian respondents report that their opinion towards social media has positively changed since the start of the pandemic, in Austria and Germany this is only reported by 9% of the respondents.

### Verification of the results of the cross-country comparison in the random theoretic sample

Even in this comparatively small sample, the three countries displayed significant differences (p < .05) in all scales derived from the STAI, SVF78, PANAS, and COVID-19 questionnaires (see Table 3 & 4). Here, too, the Italian participants portray significantly higher values on the anxiety scale and show significantly higher negative affects (the results of the pairwise post-hoc tests are shown in Table 5 & 6). The Austrian and German samples do not differ significantly in this regard. With regards to stress management strategies, the same significant results as in the total sample are shown: the Italian participants show significantly lower positive but also significantly lower negative stress management strategies compared to the other two countries. Similarly, with regard to the Covid-19 scales, the same significant mean differences were found between the three countries in the random theoretical sample.

**Table 3:** Descriptive Statistics and Kruskal-Wallis Tests results for emotional state and COVID-19-scales, *N* = 252

Scales		Italy (*n* = 84)	Austria (*n* = 84)	Germany (n = 84)	*p*	*χ*^*2*^
*STAI State Anxiety*	*M*	49.46	41.43	41.33	< .001	43.64
	*MD*	49	38.50	40		
	*SD*	3.45	12.40	10.52		
*PANAS Negative Affect*	*M*	26.88	19.44	20.45	< .001	59.91
	*MD*	27	19	20		
	*SD*	4.87	5.88	7		
*Perceived Severity of COVID-19*	*M*	8.64	8.11	8.01	.035	6.70
	*MD*	9	8	8		
	*SD*	1.41	1.69	1.75		
*Perceived Risk of Disease*	*M*	8.20	8.82	9.17	.014	8.53
	*MD*	8	9	9		
	*SD*	1.76	2.40	2.22		
*Emergency measure acceptance*	*M*	68.80	70.25	66.12	< .001	17.75
	*MD*	71	73	68		
	*SD*	5.78	7.70	10.25		
*Emotional distress due to emergency measures*	*M*	20.90	14.36	15.37	< .001	74.19
	*MD*	20	12	13		
	*SD*	5.54	6.63	7.72		
*App-Usage*	*M*	1	.30	.20	< .001	102.86
	*MD*	1	0	0		
	*SD*	.54	.60	.64

**Table 4:** Descriptive Statistics and Browne-Forsythe Tests results for coping and COVID-19-scales, *N* = 252

Scales		Italy (n = 84)	Austria (n = 84)	Germany (n = 84)	*p*	*F*	*η*^*2*^
*SVF78 Positive Stress Behavior*	*M*	12.57	19.03	19.02	< .001	210.37	.63
	*MD*	12.71	18.93	18.93			
	*SD*	1.21	2.79	2.72			
*SVF78 Negative Stress Behavior*	*M*	13.04	16.66	15.34	< .001	17.63	.12
	*MD*	12.88	15.88	15.13			
	*SD*	1.86	4.74	4.69			
*Concerns related to COVID-19*	*M*	27.56	25.05	25.99	.021	3.92	.03
	*MD*	28	25	26			
	*SD*	4.21	6.37	6.73

**Table 5:** Mann-Whitney-U-Test post hoc tests, *N* = 252

Measures	group	group	*p*	*r*
*STAI State Anxiety*	Italy	Austria	< .001	.40
	Italy	Germany	< .001	.49
	Austria	Germany	.696	
*PANAS Negative Affect*	Italy	Austria	< .001	.56
	Italy	Germany	< .001	.46
	Austria	Germany	.434	
*Perceived Severity of COVID-19*	Italy	Austria	.041	.16
	Italy	Germany	.016	.19
	Austria	Germany	.723	
*Perceived Risk of Disease*	Italy	Austria	.042	.16
	Italy	Germany	.004	.22
	Austria	Germany	.527	
*Emergency measure acceptance*	Italy	Austria	< .001	.29
	Italy	Germany	.336	
	Austria	Germany	< .001	.27
*Emotional distress due to emergency measures*	Italy	Austria	< .001	.60
	Italy	Germany	< .001	.55
	Austria	Germany	.304	
App-Usage	Italy	Austria	< .001	.60
	Italy	Germany	< .001	.71
	Austria	Germany	.085	

Effect sizes: r = .10 small, r = .30 medium, r = .50 large (Cohen, 1988)

**Table 6:** Bonferroni post hoc tests, *N* = 252

Measures	group	group	*p*
*SVF78 Positive Stress Behavior*	Italy	Austria	< .001
	Italy	Germany	< .001
	Austria	Germany	1.0
*SVF78 Negative Stress Behavior*	Italy	Austria	< .001
	Italy	Germany	.001
	Austria	Germany	.099
*Concerns related to COVID-19*	Italy	Austria	.018
	Italy	Germany	.253
	Austria	Germany	.902

## Discussion

### Interpretation of the results

The Italian participants were exposed to a lot more stressors at the time the study was conducted. They were in a phase of the pandemic, in which death and infection numbers were very high and control over the spread of the virus was only to be regained by means of very restrictive lockdown measures. In the survey phase, these measures had not yet shown much effect. Physical stressors (danger to life and limb), social stressors (e.g., separation from friends and family, farewell from the deceased), and economic stressors (fear for work, loss of job) were also considerably higher than in Austria and Germany. Moreover, curfews and the associated deprivation of freedom were only in place in Italy. The severity of the pandemic, as well as the rigor of the restrictions, thus constitutes two factors strongly differing between Italians, on the one hand, and Austrians and Germans, on the other. The impacts of these factors will be described by reference to our research questions in the following section.

#### How dangerous is the virus perceived to be?

As expected, parallel to the severity of the pandemic, Italian participants assessed the COVID-19 virus as much more dangerous than this was the case in Austria and Germany. Likewise, Italian participants also portray significantly higher levels of worries associated with the virus. Presumably, because of the comparably high mortality in Italy and the related threat to life, the individual worry to fall ill with COVID-19 is higher than in Austria and Germany.

#### How high is the subjective risk of disease perceived in dependency on the emergency measures?

Despite the prevalence of the virus, the subjective risk of disease was perceived to be lower in Italy than in Austria and Germany. This could be a positive effect of the restrictive curfews. At the time of the survey, the Italian participants had already experienced 14 days of lockdown and hence had barely any opportunity to get infected with the virus outside their own household. The infection risk was probably indeed lower. The opposite was true for the German participants, for whom the lockdown had only just started and no curfews had yet been implemented. The risk of catching the virus was thus still comparatively high for this subpopulation and the subjective risk of disease was consequently assessed to be higher. In Austria, the perceived subjective risk of disease was significantly lower than in Germany. Presumably, this was because the restrictions had already been in place for a week at the time.

#### How does the acceptance of the statutory emergency measures develop and which emotions accompany the respective measures?

Concerning the acceptance of the measures, the Italian population took a middle position between Austria and Germany. The measures set by the government were generally perceived to be very effective in all three countries. This means that even the Italian participants, who had to endure the most restrictions, showed high levels of acceptance. As expected, the emotional reaction (anger, fear) was the strongest within the Italian sample. It can be assumed that the duration and the severity of the measures did not only lead to generally high levels of emotional stress, but more concretely, also contributed to worries related to financial and economic future repercussions in Italy. This is particularly problematic, as Italy is overall economically weaker than Germany and Austria, which can be seen in the lower annual income of the subpopulation, despite the same educational level. In Austria, the comparatively less restrictive measures were accepted to a high degree and caused considerably less emotional stress. This is true, despite the fact that the Austrian subpopulation was significantly younger than the Italian one, and, as it turned out in the analyses, younger people showed stronger emotional reactions in the crises and lower levels of acceptance of the measures. In the German sample, in which the statutory measures resembled the ones in Austria, the level of emotional stress was very similar to the one in the Austrian sample and likewise comparatively low. However, while there was a high degree of approval of the measures, it was lower than in the other two countries.

#### Which effects do the statutory measures have on fear and emotional wellbeing of the population?

Despite the very restrictive statutory containment measures, the Italian sample showed considerably higher anxiety values and higher negative affect than the Austria and German sample. Since – as mentioned above – the worries associated with the COVID-19 pandemic, and in particular the financial and economic worries, were higher as well, the impression arises that fear is nurtured by the anticipated negative repercussions of the strong restrictions on the already weakened economy of the country. It presumably also caused distress that, at the time of the study, the infection numbers in Italy were very high, despite the restrictive measures. The country thus faced overall a dramatic crisis.

#### Which coping strategies can become effective under the respective measures?

Highly emotionally stressed due to the restrictive measures, Italians could draw comparatively less on positive stress processing strategies, according to our study. In SVF78 they score the lowest values on the scale *Positive stress behavior.* However, this is also due to the severe restrictive measures: the subscales of *Positive stress behavior: Diversion*, *compensatory satisfaction*, *situation control* and *Social support* measure strategies that are difficult to implement under these circumstances. The comparatively high usage of mental health apps can be interpreted as an attempt of self-stabilization by means of stress management in the sense of a badly needed distraction and compensatory satisfaction, but also as a need for social support. The markedly positive change in attitudes towards social media indicates the importance of this medial mediated social compensation in coping with the crisis. At first sight, it might seem odd that negative *Stress processing* mechanisms – which lead to a short-term stress reduction, but increase the stress burden long-term – are also lower in the Italian sample than in the other two countries. However, the individual scales show, that the negative stress processing strategies *Avoidance* and *Escape tendency* could not be implemented due to the restrictions and that there was hardly any reason for *Self-accusation* in a pandemic, in which control was lost over the virus and thus contagion changes could no longer be traced. The *Perseveration* strategy was potentially lower due to a “numbness” setting in after 14 days of lockdown, as opposed to Austrians and Germans, for which the drama of the happenings was comparatively new, as they were still at the beginning of the lockdown, i.e., the beginning of the phase in which governments had to counteract as control seemed to slip away.

### Summary of the results and implications

Overall, the Italian population perceived the virus as comparatively more dangerous. This corresponded to the pandemic situation within the country, at the time of the study. However, the subjective risk of disease was judged to be lower, as restrictive emergency measures went hand in hand with a lower risk of infection. At the same time, the emotional stress associated with the very restrictive lockdown conditions, practically equaling confinement to the own apartment, was reported to be much higher. Additionally, it became apparent that the measures did not alleviate the prevalent fear of the population. The stricter measures also prevented the application of many stress processing strategies such as diversion, compensatory satisfaction, and social support, which, in turn, fostered the preservation of stresses and fear. Our study thus shows through the comparison of countries with restrictive and moderate lockdown measures, that such massive constraints to the containment of the virus may make sense from an epidemiological point of view, but exceeds the coping capabilities of the population and do not reduce fear. The opposite is true: Such massive constraints to freedom contribute to additional stress, which manifests itself in our study in considerably increased negative affect. Consequently, it is in accordance with the bio-medical-psychosocial model, also necessary during pandemics [28] to reconcile epidemiological protective strategies with psychosocial coping capabilities in the best way possible. This means, that necessary lockdowns should be designed in a way, that does not overwhelm the emotional stress capabilities of the population. Otherwise, the consequence can be expected to be highly prevalent secondary diseases due to stress. It has been shown that during the lockdown the number of mental disorders has quadrupled [29] and suicide rates have increased enormously.

Current estimates assume that there will be between 2135 and 9570 additional suicides worldwide in the context of the COVID-19-pandemic [30].

The same problems are reflected in the increase of digital media usage such as online gaming or social media. Though, the opportunity to digitally exchange with family and friends during the lockdown and thus to experience social support without social-physical presence or simply to receive entertainment, proved to be a constructive coping strategy [31, 32], studies have shown that addictive media usage increased during the lockdown [29]. This is not surprising when digital communication and instrumental media usage for emotion regulation, remain one of the last possibilities to satisfy socio (physical) needs.

### Methodological limitations

The strongest limitations of this study result from online recruitment (forums and social media platforms) applying the snowball sampling procedure. This non-probability sampling technique may be reflected in a bias resulting from the self-selection of participants whose central characteristics do not correspond to the German and Austrian populations. The sample in this study, for instance, has a higher proportion of women with a high level of education. At the same time, as data obtained from self-assessment does not constitute an accurate representation of behaviors, statements about the actual compliance with emergency measures require caution. However, since the survey was anonymous, we can presume a low social desirability tendency, although recall biases may influence self-assessment.

Further, the study at hand constitutes a cross-sectional study. It does thus not allow for statements on, for example, whether or not emotional experience and strain change throughout the pandemic in general, and between the first and further lockdowns in particular, and if so how. Likewise, we have not collected any clinical information on the existence of mental illnesses or certain physical illnesses, considered risk factors for a severe course of a Covid-19 infection.

### Research outlook

Our findings should be replicated in further studies with a representative sample. Qualitative interviews could capture the emotional wellbeing of the population groups, which have experienced different degrees of severity of the pandemic, in-depth. A quantitative analysis of media coverage and the information policy of the respective governments could help to understand, for example, why there is a higher approval of the measures in Austria than in Germany, despite the measures being very similar in content. While studies have already proven the influence of information consumed via social networks on levels of anxiety and stress (for an overview see [33]), no study has yet contrasted countries subjected to varying degrees of the pandemic. Likewise, further studies on this question should collect the above-mentioned clinical information as it might influence the results. I.e., people subjected to physical or mental risk factors could experience a different extent of fear, emotional strain, and negative affects.

## Supplementary Information

**Additional file 1.**

## Data Availability

The datasets used and/or analyzed during the current study are available from the corresponding author on reasonable request.
