# Supplementary material for: Emotional distress in the early stages of the COVID-19 related lockdowns depending on the severity of the pandemic and emergency measures: a comparative online-survey in Germany, Austria and Italy
Source: BMC Psychiatry. 2021 Oct 15;21:509. doi: 10.1186/s12888-021-03505-7 (PMC8518266; doi:10.1186/s12888-021-03505-7)
Supplement: Supplementary file 1 — Additional file 1. [file 12888_2021_3505_MOESM1_ESM.docx]

**Supplementary Information**

**Emotional distress in the early stages of the COVID-19 related lockdowns depending on the severity of the pandemic and emergency measures: A comparative online-survey in Germany, Austria and Italy**

Christiane Eichenberg^1*^, Martin Grossfurthner^2^, Sibylle Kietaibl^3^, Greta Riboli^2,4^, Rosita Borlimi^4^ & Stefana Holocher-Benetka^2^

^1^ Institute of Psychosomatics, Faculty of Medicine, Sigmund Freud University, Vienna, Austria

^2^ Faculty of Psychology, Sigmund Freud University, Vienna, Austria

^3^ Faculty of Medicine, Sigmund Freud University, Vienna, Austria: Evangelical Hospital Vienna, Austria

^4^ Faculty of Psychology, Sigmund Freud University, Milano, Italy

**APPENDIX**

1. **Translation of the Covid19-pandemic questionnaire**

*Perceived Severity* *of Covid19* (2 items):

Please give your opinion on the following scales:

The coronavirus is…

| harmless | O | O | O | O | O | dangerous |
| --- | --- | --- | --- | --- | --- | --- |
| comparable to influenza | O | O | O | O | O | more dangerous than influenza |

*Perceived risk of disease* (3 items):

| To what extent you are susceptible of catching COVID-19? | | | | | | |
| --- | --- | --- | --- | --- | --- | --- |
| not at all | O | O | O | O | O | great extent |
| To what extent you believe that you are able to avoid an infection with COVID-19? | | | | | | |
| not at all | O | O | O | O | O | great extent |
| To what extent you believe that you are a risk factor for transmitting the infection? | | | | | | |
| not at all | O | O | O | O | O | great extent |

*Emotional distress due to emergency measures* (10 items):

Please give your opinion on these measures:

| Self-isolation due to coronavirus… | | | | | | |
| --- | --- | --- | --- | --- | --- | --- |
|  | not at all |  | partly |  | a lot |  |
| makes me angry | O | O | O | O | O |  |
| frightens me | O | O | O | O | O |  |
|  |  |  |  |  |  |  |
| Quarantine due to coronavirus… | | | | | | |
| makes me angry | O | O | O | O | O |  |
| frightens me | O | O | O | O | O |  |
|  |  |  |  |  |  |  |
| Traveling restrictions due to coronavirus… | | | | | | |
|  | not at all |  | partly |  | a lot |  |
| makes me angry | O | O | O | O | O |  |
| frightens me | O | O | O | O | O |  |

| Smart working due to coronavirus… | | | | | | |
| --- | --- | --- | --- | --- | --- | --- |
| makes me angry | O | O | O | O | O |  |
| frightens me | O | O | O | O | O |  |

| Cancellation of events (exhibitions, sports, concerts, cinema) due to coronavirus… | | | | | | |
| --- | --- | --- | --- | --- | --- | --- |
| makes me angry | O | O | O | O | O |  |
| frightens me | O | O | O | O | O |  |

*Emergency measures acceptance* (15 items):

Please give your opinion on these measures:

| Self-isolation due to coronavirus… | | | | | | |
| --- | --- | --- | --- | --- | --- | --- |
|  | not at all |  | partly |  | a lot |  |
| is reasonable | O | O | O | O | O |  |
| can prevent spread | O | O | O | O | O |  |
| is exaggerated | O | O | O | O | O |  |
|  |  |  |  |  |  |  |
| Quarantine due to coronavirus… | | | | | | |
|  | not at all |  | partly |  | a lot |  |
| is reasonable | O | O | O | O | O |  |
| can prevent spread | O | O | O | O | O |  |
| is exaggerated | O | O | O | O | O |  |
|  |  |  |  |  |  |  |

| Traveling restrictions due to coronavirus… | | | | | | |
| --- | --- | --- | --- | --- | --- | --- |
|  | not at all |  | partly |  | a lot |  |
| is reasonable | O | O | O | O | O |  |
| can prevent spread | O | O | O | O | O |  |
| is exaggerated | O | O | O | O | O |  |
| Smart working due to coronavirus… | | | | | | |
|  | not at all |  | partly |  | a lot |  |
| is reasonable | O | O | O | O | O |  |
| can prevent spread | O | O | O | O | O |  |
| is exaggerated | O | O | O | O | O |  |

| Cancellation of events (exhibitions, sports, concerts, cinema) due to coronavirus… | | | | | | |
| --- | --- | --- | --- | --- | --- | --- |
|  | not at all |  | partly |  | a lot |  |
| is reasonable | O | O | O | O | O |  |
| can prevent spread | O | O | O | O | O |  |
| is exaggerated | O | O | O | O | O |  |

*Concerns related to the COVID19-pandemic* (8 items):

Using the following scale, please rate the extent to which the following concerns apply to you in times of Corona:

|  | not at all |  | partly |  | a lot |
| --- | --- | --- | --- | --- | --- |
| I am concerned about my own health | O | O | O | O | O |
| I am concerned about the health of my relatives/friends | O | O | O | O | O |
| I am concerned about my financial situation | O | O | O | O | O |
| I am concerned about people having to suffer because of Corona | O | O | O | O | O |
| I am concerned about people dying because of the coronavirus | O | O | O | O | O |
| I am concerned about whether society is recovering from this crisis | O | O | O | O | O |
| I am concerned about the economic impact | O | O | O | O | O |
| I am concerned that medical care will no longer be available for other health problems | O | O | O | O | O |

*E-mental health usage* (4 items):

To better manage psychosocial stress due to coronavirus I use apps (multiple answers possible):

|  | yes | no |
| --- | --- | --- |
| I am using apps for overcoming my fears | O | O |
| I am using apps to keep in contact with others | O | O |
| I am using apps to cope with stress | O | O |
| I am using apps for distraction | O | O |

The situation due to the coronavirus has changed my opinion about social media…

|  | yes | no |
| --- | --- | --- |
| yes, to the positive | O | O |
| yes, to the negative | O | O |
| no, not changed | O | O |
